# Computerized adaptive testing of population psychological distress: simulation-based evaluation of GHQ-30

**DOI:** 10.1007/s00127-015-1157-4

**Published:** 2015-12-21

**Authors:** Jan Stochl, Jan R. Böhnke, Kate E. Pickett, Tim J. Croudace

**Affiliations:** Department of Health Sciences, University of York, Area 4, ARRC Building, York, YO10 5DD UK; Hull York Medical School (HYMS), University of York, Area 4, ARRC Building, York, YO10 5DD UK; School of Nursing and Health Sciences, University of Dundee, 11 Airlie Place, Dundee, DD1 4HJ UK; Department of Psychiatry, University of Cambridge, Herchel Smith Bldg, Robinson Way, Cambridge, CB2 0SZ UK

**Keywords:** Computerized adaptive testing, Item response theory, Bifactor model, Measurement invariance, General Health Questionnaire

## Abstract

**Purpose:**

Goldberg’s General Health Questionnaire (GHQ) items are frequently used to assess psychological distress but no study to date has investigated the GHQ-30’s potential for adaptive administration. In computerized adaptive testing (CAT) items are matched optimally to the targeted distress level of respondents instead of relying on fixed-length versions of instruments. We therefore calibrate GHQ-30 items and report a simulation study exploring the potential of this instrument for adaptive administration in a longitudinal setting.

**Methods:**

GHQ-30 responses of 3445 participants with 2 completed assessments (baseline, 7-year follow-up) in the UK Health and Lifestyle Survey were calibrated using item response theory. Our simulation study evaluated the efficiency of CAT administration of the items, cross-sectionally and longitudinally, with different estimators, item selection methods, and measurement precision criteria.

**Results:**

To yield accurate distress measurements (marginal reliability at least 0.90) nearly all GHQ-30 items need to be administered to most survey respondents in general population samples. When lower accuracy is permissible (marginal reliability of 0.80), adaptive administration saves approximately 2/3 of the items. For longitudinal applications, change scores based on the complete set of GHQ-30 items correlate highly with change scores from adaptive administrations.

**Conclusions:**

The rationale for CAT-GHQ-30 is only supported when the required marginal reliability is lower than 0.9, which is most likely to be the case in cross-sectional and longitudinal studies assessing mean changes in populations. Precise measurement of psychological distress at the individual level can be achieved, but requires the deployment of all 30 items.

## Introduction

Goldberg’s General Health Questionnaire (GHQ) [1] items have been used frequently by population and health service researchers for measuring levels of clinically significant but non-specific psychological distress. Tens of thousands of survey respondents and patients from a variety of populations and health care settings have completed one of the four available versions with 12, 28, 30 or (rarely) 60 items [2, 3]. Simple scoring methods and cut-off scores for “caseness” are commonly applied and such practice has supported a large volume of studies.

A range of psychometric and technological developments have taken place in educational, social survey and clinically oriented assessment research over recent decades. Among the most important are those that allow for some aspect of personalization, especially if these can be aligned to methods that are efficient, reduce burden, and appeal to respondents. Additionally, from a “psychometric epidemiology” [2] perspective two aspirations remain: (1) to integrate what can be known about individuals or populations from items across versions and (2) how to apply the item set in a manner that does not rely on the “legacy” or fixed-length versions [4]. In this paper, we address the second aspect by providing a full demonstration of the computerized adaptive testing (CAT) paradigm [5] as it might be adopted for GHQ-30 data or other item pools: to personalize assessments, make them more efficient, and tailor them in length and administration to the mode needed for a specific implementation (e.g., pencil and paper, mobile device, desktop computer).

Although CAT originated in educational settings where the target for measurement would typically be an examinee’s ability level, our exposition here is in the wider setting of population health, social science, or epidemiological and lifestyle surveys. CAT is an approach involving computer-based administration of questionnaires using principles able to adapt the content to the score level of the person. Such adaptation is based on the concept of item information introduced in item response theory (IRT) modelling. Specifically, CAT algorithms will select and administer the most informative items for each respondent based on (1) known item characteristics obtained from prior calibration using IRT models and (2) on what is known about an individual’s level of the measured attribute (construct) from their responses to previous questions. In CAT, the required level of measurement accuracy for the target construct is usually fixed instead of fixing the number of items as in the traditional approach. CAT then selects optimal item sequences until this goal is met. As a result, typically fewer items are administered and each respondent encounters a unique set of items, with the potential benefit that the questions presented might seem more relevant to the respondent, since they are targeted closer to their distress level. These two features are synergistic, hence they result in improved efficiency [6].

CAT principles have been successfully applied in mental health assessment [6–8] and were found to outperform traditional static tests [9]. However, the increase in efficiency may in specific contexts not be sufficient to justify the added technical requirements for CAT administration [9]. Fortunately, recent developments and availability of open-source CAT algorithms [10–13] make its implementation easier and less costly.

The aim of our study was to evaluate the potential of CAT for the GHQ-30 item pool and to demonstrate the steps required for transition from the fixed-length test to an adaptive version, which are generally agreed [14, 15]. For this purpose, we used data collected with traditional methods (i.e. paper and pencil self-completion). The structure of the study was as follows: We first followed an established approach [14] to estimate the IRT parameters to evaluate model fit and to derive psychometric properties of items (i.e. calibrate the item pool). Building on these results, we aimed to contribute further detail on a more complex scenario: repeated adaptive administration in longitudinal studies. For this we examined how a CAT version of GHQ-30 could be used to measure change in psychological distress. We begin, however, with the usual case of a single GHQ-30 administration as is applicable to a cross-sectional study.

## Methods

### General Health Questionnaire (30 item version)

Goldberg’s General Health Questionnaire [1] items are typically deployed in one of four paper forms as self-completion questionnaires comprising 12, 28, 30 and 60 items. In the context of CAT feasibility evaluation, it makes sense to consider the items as a set (“item bank”) rather than any subset of items, per se. However, the 60 item version is rarely applied in current survey research and therefore no existing large enough dataset was available for analysis. The largest set in common use is the GHQ-30 [16]. The GHQ-30 was developed as a shortened version of the GHQ-60, intentionally avoiding somatic items, but retaining the principle dimension of general psychological distress. The responses for all GHQ-30 items are captured on four verbally anchored categories typically scored consecutively from 1 to 4, where higher scores indicate more distress. An important feature of the GHQ-30 is its inclusion of an equal number of positively and negatively phrased items that have slightly different verbal anchors for their response categories. This feature has led to a debate about a so-called “methods” factor causing differential response behaviour between those two item sets and it is sometimes addressed in psychometric modelling, for example using a bifactor model [17–20].

### Population sample for empirical item analysis: Health and Lifestyle Survey (HALS)

The Health and Lifestyle Survey was designed to examine the distribution of, and the relationship between, physical and mental health, health-related behaviour and social circumstances in adults of all ages and circumstances living in their own homes in all parts of Great Britain [21].

Datasets of GHQ-30 responses were taken from two waves of the HALS study [21, 22]; in wave one (baseline), a total of 9003 adults (43.4 % males, 56.4 % females) aged 18 and over (mean age = 45.9, SD = 17.7), living in private households in Great Britain in 1984–1985 were recruited and complete GHQ-30s were obtained from *n*_1_ = 6317 individuals. The same sample was surveyed again 7 years later (5352 adults, 43.0 % males, 57.0 % females, mean age = 51.8, SD = 16.0) where complete GHQ-30s were obtained from *n*_2_ = 3779 participants. Only respondents who participated in both waves and provided complete GHQ-30 at both occasions were analysed in this study (*n* = 3445).

### Steps of CAT development for measurement of change

#### Step 1: IRT calibration of the GHQ-30 item bank

If the aim is to migrate a paper and pencil questionnaire to an adaptive version, the number of latent factors underpinning item responses must first be assessed and a clear understanding be obtained regarding how the items of the questionnaire relate to these factors. These questions can be answered through the assessment of fit of various factor analytic models. While fitting unidimensional models is straightforward, more complicated multidimensional structures are often required to fit the data. Such multidimensionality can be of two kinds: between-item, where each item loads on a single factor only, and within-item, where each item loads on multiple factors [23]. In case of the former, the traditional approach is to calibrate each cluster of items separately. In the case of the latter, the researcher needs to obtain estimates using specialized software such as MPlus [24] or using R packages *mirt* [25] or *lavaan* [26] and subsequently converts estimates into IRT parameters.

**Fig. 1:**
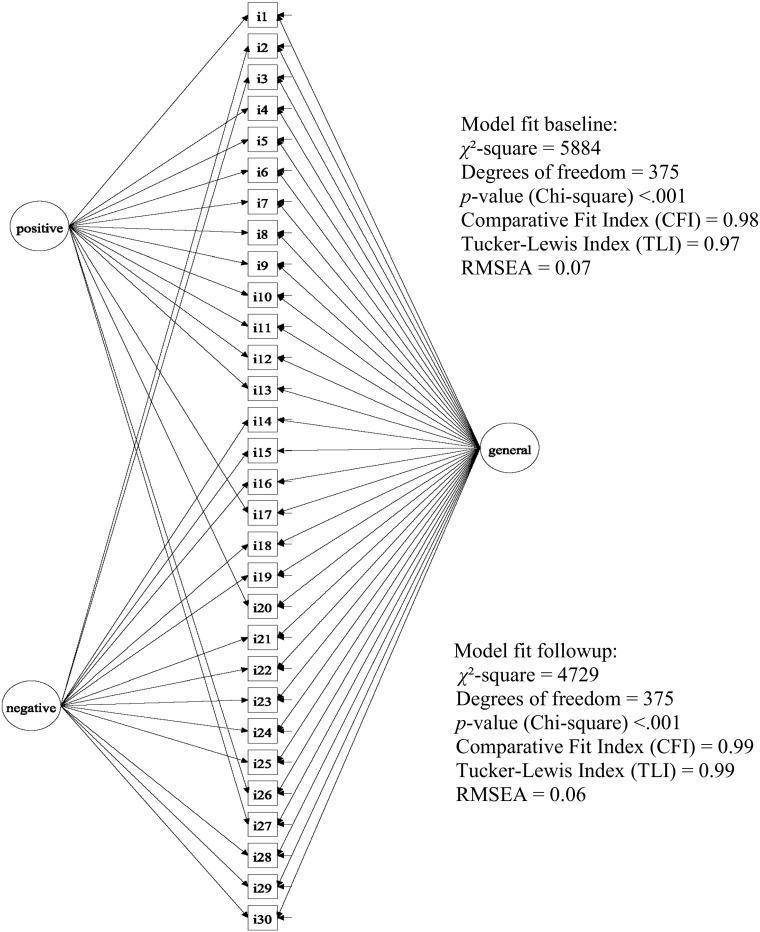
Bifactor model for GHQ-30 items at baseline and follow-up

Here, we consider a more complex structure, which is consistent with a multidimensional (within-item) approach: we assume, a priori, that all GHQ-30 items contribute mainly to the measurement of a single latent dimension of “psychological distress”. In addition to this dominant (general) factor, responses might also be influenced by methodological features such as item wording (positive and negative item wording). Several approaches have been suggested to model variance specific to methods factors [27, 28] from which we chose to apply a bifactor model (see Fig. 1).^1^

Since the dataset contained a repeat GHQ-30, we desired a common model for the baseline and follow-up data. We achieved this by specifying a structural equation model for categorical items and estimated this model in *lavaan*, both for the baseline and follow-up data. Mean and variance adjusted weighted least square (WLSMV) was used to estimate the bifactor model parameters. At this stage, the researcher’s primary interest focuses not so much on estimates (factor loadings, thresholds) but rather aims to assess model fit (though brief checking of estimates is desirable—for example to detect improper solutions such as Heywood cases [29]). The suitability of our model was confirmed via evaluation of several fit indices (see Fig. 1) which showed a reasonable model fit for both occasions when estimating individual sets of parameters for each occasion [30, 31].

When instruments are used across multiple sub-populations or longitudinally, the issue of differential item functioning (DIF) needs to be addressed. The main aim of DIF analysis is to test whether the item characteristics are the same across sub-populations or remain unchanged over time. Absence of DIF allows comparisons of distributions of latent scores across populations. If DIF is present and ignored, estimation of change over time might be biased.

General methods for assessment of DIF include ordinal regression and invariance of IRT parameters. For the GHQ-30 we used iterative hybrid ordinal logistic regression approach available in R library *lordif* [32]. Given the relatively large sample size, pseudo-*R*^2^ (change ≥0.02) was used as a criterion for DIF detection [33]. Three GHQ-30 items were flagged to show DIF (item 16: “Found life a struggle”, pseudo-*R*^2^ = 0.030; item 19: “Scared or panicky”, pseudo-*R*^2^ = 0.03; item 25: “Felt life hopeless”, pseudo-*R*^2^ = 0.05).

In summary, the first step showed that the GHQ-30 can be described largely by a single dimension and apart from three items the GHQ-30 was also invariant across time (DIF). These items needed special attention in the simulation study as described below.

#### Step 2: evaluation of GHQ-30-based CAT assessment

The aim of this step was to obtain stable IRT parameters from our factor analyses (above) that could be used as input parameters for running a CAT simulation to evaluate the adaptive administration of this item bank. For a single population and cross-sectional data this can be done by obtaining the model parameters from a well-fitting model. If multiple populations or longitudinal assessments are the basis for the calibration, with the exception of the DIF items all item parameters need to be constrained over population/time points to establish measurement invariance. For bifactor models, general and method factor loadings need to be constrained as well as item thresholds. Items with constrained parameters serve as “anchors” to make latent scores comparable over time or populations. Obviously parameters of DIF items are not constrained: this is how DIF is addressed. In our case for the three DIF items the individually estimated parameters for baseline/follow-up model were used. To summarize this step, we estimated a categorical data bifactor CFA model with WLSMV estimation using both baseline and follow-up data, and constrained loadings and thresholds to be equal over time for all but the three DIF items. Model fit for this constrained model was still acceptable (CFI = 0.93, TLI = 0.93, RMSEA = 0.07).

When the parameters are estimated in SEM software they need to be converted into IRT parameters using the following formulae [34, 35]; for each item *i* = 1,…,*M* influenced by *p* = 1,…,*P* factors, the discrimination ($\alpha_{ip}$) and *k* IRT thresholds ($t_{ik}$) on item *i* are

$$\alpha_{ip} = \frac{{1.7 \times \lambda_{ip} }}{{\sqrt {1 - \sum\nolimits_{p = 1}^{P} {\lambda_{ip}^{2} } } }}\;{\text{and}}\;t_{ik} = \frac{{1.7 \times \tau_{ik} }}{{\sqrt {1 - \sum\nolimits_{p = 1}^{P} {\lambda_{ip}^{2} } } }},$$

where $\lambda_{ip}$ is factor loading of the item on factor *p*, $\tau_{ik}$ are the corresponding item thresholds and the scaling constant 1.7 converts estimates from the normal ogive metric of the factor model into logistic IRT metric needed for the CAT application. In the case of our bifactor model considered for the GHQ-30, each item loaded on the general (distress) factor as well as one method factor (positive or negative) and therefore *P* = 2. As noted previously, to eliminate the influence of item wording, we only considered and converted IRT estimates for the general factor. Converted IRT estimates of GHQ-30 items for baseline and follow-up are in Table 1. Note that with the exception of the DIF items (item 16, 19 and 25), item parameters are the same for baseline and follow-up.

**Table 1:** IRT estimates of GHQ-30 items (in logistic metric)

Item #	Item stem	Baseline				Follow-up			
		Discrimination	Threshold 1	Threshold 2	Threshold 3	Discrimination	Threshold 1	Threshold 2	Threshold 3
1	Could concentrate	1.01	−3.42	1.88	3.99	id	id	id	id
2	Lost sleep	1.38	−0.41	2.74	5.06	id	id	id	id
3	Restless nights	0.43	−0.43	0.89	1.82	id	id	id	id
4	Busy or occupied	0.38	−1.41	2.56	3.95	id	id	id	id
5	Out of the house	0.51	−1.84	1.99	3.53	id	id	id	id
6	Managing well	0.68	−1.36	3.51	4.96	id	id	id	id
7	Doing things well	1.30	−3.02	3.91	6.86	id	id	id	id
8	Satisfied with task	1.24	−2.84	3.84	6.76	id	id	id	id
9	Feel warmth and affection	0.44	−1.47	2.69	4.17	id	id	id	id
10	Get on with others	0.59	−2.41	3.27	5.09	id	id	id	id
11	Chatting with others	0.43	−1.70	2.30	3.99	id	id	id	id
12	Playing a useful part	0.82	−2.16	2.36	4.16	id	id	id	id
13	Capable make decisions	0.54	−1.60	1.77	3.26	id	id	id	id
14	Felt under strain	1.93	−1.39	2.19	5.12	id	id	id	id
15	Could not overcome difficulties	2.05	−0.41	3.18	5.30	id	id	id	id
16	Found life a struggle	0.81	−0.62	2.02	3.21	3.29	−1.27	4.14	7.86
17	Enjoying activities	0.62	−1.73	1.20	2.28	id	id	id	id
18	Taking things hard	1.71	−0.87	2.46	4.69	id	id	id	id
19	Scared or panicky	1.02	0.13	2.55	3.74	2.54	0.20	4.05	6.63
20	Face problems	0.98	−2.67	2.75	4.42	id	id	id	id
21	Felt everything on top	2.96	−0.66	3.50	7.14	id	id	id	id
22	Unhappy and depressed	2.87	−0.20	2.99	6.05	id	id	id	id
23	Lost confidence	2.96	0.34	3.67	6.58	id	id	id	id
24	Felt worthless	2.83	1.84	4.61	6.75	id	id	id	id
25	Felt life hopeless	1.37	0.97	2.70	4.48	1.81	1.10	3.08	4.10
26	Hopeful about future	0.90	−1.66	2.21	3.74	id	id	id	id
27	Feeling happy	0.72	−1.60	1.65	2.94	id	id	id	id
28	Nervous and strung up	2.62	0.46	3.69	6.63	id	id	id	id
29	Felt life not worth living	2.78	3.02	5.40	7.10	id	id	id	id
30	Nerves too bad	2.35	2.65	5.02	6.55	id	id	id	id

Slightly modified item stems taken from [16]

*id* parameter is identical to the corresponding one at baseline

### CAT simulation

There are two ways of conducting a CAT simulation study: (a) a matrix of item parameter estimates from the IRT calibration is available as well as a matrix of item responses (observed or simulated) which can then be simultaneously processed during simulation. In such case, the simulation enables a researcher to evaluate the efficiency of the CAT approach in comparison with the traditional administration of the full set of items; (b) a vector of true latent psychological distress score values of person (*θ*_s_) can be provided instead of actual responses to items from a completed study. Then, the simulation can be used to evaluate the efficiency of CAT administration with respect to these true latent distress score values.

We used IRT parameters from the model reported in Table 1 and real item responses to GHQ-30 from the HALS study; that is we adopted method (a) from the previous paragraph. Beyond evaluating the person–item match [1], our simulation setup compared different estimators (how the latent score is determined) and different item selection methods (how the next item is chosen). CAT simulations were performed using *catIrt* [10] package in R.

We used three different estimators, two item selection methods and two prior distributions in our simulation study (listed in “Appendix”). We used this design to evaluate whether combinations would have a differential effect on the administration procedure in this specific case and to shed more light on the question of whether these methods differed in practically relevant ways. Specifically, maximum likelihood estimation (MLE), Bayesian modal estimation (BME), and expected a priori estimation (EAP) were our three choices for latent score (*θ*) estimation. BME and EAP estimators allow for prior distributions to be specified—a useful feature when knowledge or hypotheses about the latent construct distribution in the target population are available; in this study we considered uniform and standard normal. Finally, unweighted Fisher information (UW-FI) and pointwise Kullback–Leibler divergence (FP-KL) (see [10] for details) were two item selection methods we examined. A list of settings of our simulation study is provided in “Appendix” (further details and simulation code in R are available from the corresponding author) and options available in the *catIrt* package from the reference manual [10]. The simulated CAT administration was set to end when (a) the preset precision for each simulee was reached (cutoff values provided in “Appendix”) or (b) when all 30 items were administered.

We first evaluate the results by the average number of items administered to reach the desired termination criteria. In HALS, due to the two-waves of GHQ responses, we report this for both baseline and follow-up (in Table 2). The results indicated that, to achieve a high level of reliability^2^ [36–38] for a latent construct score (>0.9), almost all GHQ items need to be administered. This result held regardless of the method of *θ* estimation or item selection algorithm chosen. In a simulation scenario relevant to those who would accept a moderate level of reliability (in between levels of 0.8 and 0.9), CAT administration was shown to offer the potential to reduce the number of test items to half (by administering only 15 of the GHQ-30 item set when the desired reliability cutoff is 0.84). If the study design can accommodate an even lower level of reliability then the results revealed that around ten items are required (in effect eliminating the need to administer two-thirds of the GHQ-30 items). This result was achieved when a reliability cutoff of 0.80 was specified in CAT.

**Table 2:** Average number of administered items over 3445 simulated CAT administrations and (in brackets) the proportion of CAT administrations which reached the corresponding marginal reliability

*θ* estimator	Item selection	Prior	Marginal reliability (baseline)						
			0.96	0.94	0.91	0.88	0.84	0.8	0.75
MLE	UW-FI	–	30 (0 %)	30 (0.1 %)	28 (11.4 %)	20 (63.5 %)	13 (87.5 %)	10 (94.6 %)	8 (97.6 %)
MLE	FP-KL	–	30 (0 %)	30 (0.1 %)	28 (11.4 %)	20 (63.5 %)	14 (87.5 %)	10 (94.7 %)	8 (97.6 %)
BME	UW-FI	Uniform	30 (0 %)	30 (0.1 %)	28 (10.1 %)	21 (60.1 %)	15 (83.5 %)	11 (90.7 %)	9 (93.6 %)
BME	FP-KL	Uniform	30 (0 %)	30 (0.1 %)	28 (10 %)	21 (60.2 %)	15 (83.5 %)	11 (90.7 %)	9 (93.6 %)
BME	UW-FI	Normal	30 (0 %)	30 (0.9 %)	27 (26.6 %)	16 (82.1 %)	9 (97.2 %)	6 (99.4 %)	4 (100 %)
BME	FP-KL	Normal	30 (0 %)	30 (0.9 %)	27 (26.6 %)	16 (82.1 %)	9 (97.2 %)	6 (99.4 %)	4 (100 %)
EAP	UW-FI	Uniform	28 (7.6 %)	27 (9.8 %)	26 (15.2 %)	20 (60.5 %)	14 (87 %)	10 (95 %)	8 (98.3 %)
EAP	FP-KL	Uniform	28 (7.4 %)	27 (9.6 %)	26 (15.1 %)	20 (60.2 %)	14 (87 %)	10 (95 %)	8 (98.3 %)
EAP	UW-FI	Normal	30 (0.6 %)	30 (1.5 %)	28 (18.4 %)	17 (82 %)	10 (97 %)	7 (99.4 %)	5 (100 %)
EAP	FP-KL	Normal	30 (0.6 %)	30 (1.5 %)	28 (18.4 %)	17 (81.9 %)	10 (97.1 %)	6 (99.4 %)	5 (100 %)

*θ* estimator	Item selection	Prior	Marginal reliability (follow-up)						
			0.96	0.94	0.91	0.88	0.84	0.8	0.75
MLE	UW-FI	–	30 (0 %)	30 (3.5 %)	26 (30.5 %)	16 (78.3 %)	10 (95.2 %)	8 (97.7 %)	6 (98.5 %)
MLE	FP-KL	–	30 (0 %)	30 (3.5 %)	26 (30.6 %)	16 (78.4 %)	10 (95.3 %)	8 (97.7 %)	6 (98.5 %)
BME	UW-FI	Uniform	30 (0 %)	30 (2.2 %)	27 (26.5 %)	18 (72.2 %)	12 (89.1 %)	10 (91.5 %)	8 (92.4 %)
BME	FP-KL	Uniform	30 (0 %)	30 (2.2 %)	27 (26.4 %)	18 (72 %)	12 (89.1 %)	10 (91.5 %)	8 (92.4 %)
BME	UW-FI	Normal	30 (0 %)	29 (4.7 %)	23 (50.2 %)	12 (94.1 %)	7 (99.1 %)	5 (99.7 %)	4 (100 %)
BME	FP-KL	Normal	30 (0 %)	29 (4.7 %)	23 (50.2 %)	12 (94.1 %)	7 (99.1 %)	5 (99.7 %)	3 (100 %)
EAP	UW-FI	Uniform	27 (10.3 %)	27 (12.8 %)	25 (27.1 %)	17 (74.7 %)	11 (93.7 %)	8 (98 %)	7 (99.1 %)
EAP	FP-KL	Uniform	27 (10.5 %)	27 (12.8 %)	25 (27 %)	17 (74.4 %)	11 (93.6 %)	8 (97.9 %)	7 (99.1 %)
EAP	UW-FI	Normal	30 (1.1 %)	29 (6.1 %)	24 (46.5 %)	13 (93.1 %)	8 (98.7 %)	6 (99.7 %)	4 (99.9 %)
EAP	FP-KL	Normal	30 (1.1 %)	29 (6.1 %)	24 (46.4 %)	13 (93.1 %)	8 (98.7 %)	6 (99.7 %)	4 (99.9 %)

*MLE* maximum likelihood, *BME* Bayesian modal estimation, *EAP* expected A-posteriori estimation, *UW-FI* unweighted Fisher information, *FP-KL* pointwise Kullback–Leibler divergence

In Table 2, we also report the percentage of CAT administrations which reached the desired level of measurement precision. The numbers mirror the difficulty to reach high reliabilities (>0.90) with the GHQ-30 item bank, but for lower reliabilities a substantial share of the simulated assessments was above the preset cutoff. The EAP estimator with uniform prior seemed to be slightly superior but only for very high levels of measurement precision.

Some comment on the effect of the selected estimation method is also warranted: as expected, Maximum likelihood-based and Bayesian-based *θ* estimators with non-informative (uniform) priors appeared to be similarly effective (in fact MLE and BME with uniform prior are formally equivalent); however, the results show that choosing a normal prior distribution did contribute to greater efficiency of administration, which was evidenced by a reduction in the number of administered items. Informative (normal) priors helped to decrease the number of items even further. As a final nuance, we could also see from the scope of our current simulation evidence that information-based and Kullback–Leibler-based item selection algorithms are equally effective in this regard.

The final comment relates to the comparison of the simulation for followup versus baseline data. Interestingly, the number of administered items was slightly lower for the follow-up GHQ data. This was a direct result of larger discrimination parameters evident in the second IRT calibration for the three items for which longitudinal DIF was detected.

**Fig. 2:**
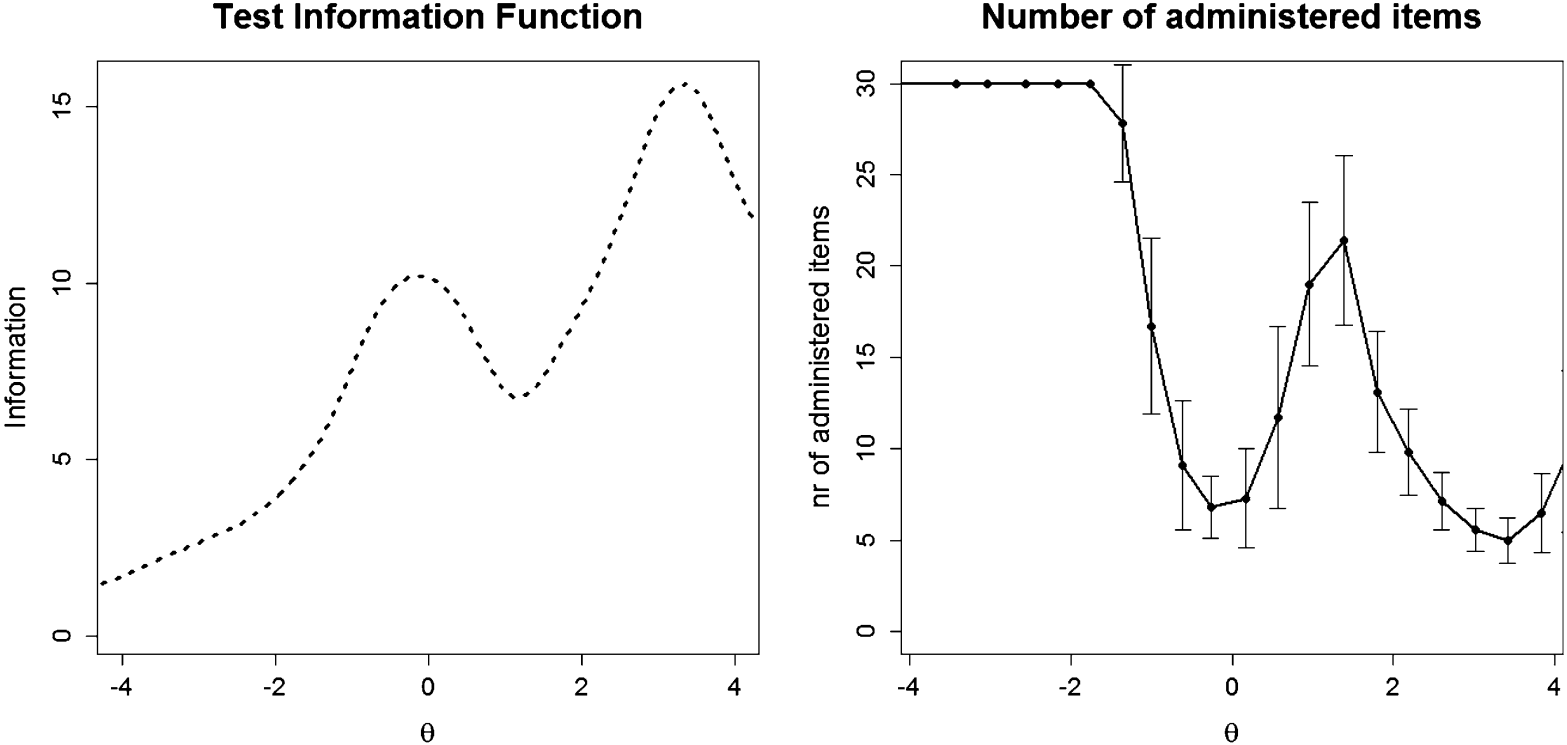
Relationship between trait levels and test information (*left*) and trait levels and number of administered items to reach the reliability cutoff of 0.84 (*right*) in CAT administration mode over 3445 simulated CAT administrations using MLE as theta estimator and UW-FI for item selection. *Whiskers* depict corresponding standard deviations. Higher values of *θ* indicate higher levels of distress

The number of items that need to be administered was not constant across the range of possible *θ* values but was related to information available along the measured continuum. Figure 2 provides a plot allowing a more detailed understanding of this patterned relationship. The left panel of Fig. 2 shows how the test information function depends on the latent trait level. Higher values in this graph indicate latent trait ranges (*x*-axis) where higher precision/smaller standard errors were achieved. Clearly, from this graph the GHQ-30 was most informative for respondents with higher levels of distress (“0” on the *x*-axis representing the population mean across both administrations). The right panel shows the mean number of administered items depending on the trait level. Especially for low levels of distress a high number of items has to be deployed, which underlines the importance of population targeting in CAT administration: the larger the share of respondents with low distress levels, the larger the share of respondents for whom all items will be administered, potentially even without reaching the desired level of reliability.

### Measurement of change

Change in psychological distress can be measured by

$$\theta_{\text{change}} = \theta_{\text{followup}} - \theta_{\text{baseline}}$$

where $\theta_{\text{baseline}}$ and $\theta_{\text{followup}}$ are IRT-based *θ* estimates on general factor for each person based on administration of whole set of GHQ-30 items. Alternatively, GHQ-30 can be administered using CAT at both occasions. This approach introduces another way of exploiting CAT, when there are a larger number of items in the item pool. Table 3 shows the correlation coefficients between $\theta_{\text{change}}$ estimates when all items of GHQ-30 are administered and the CAT alternative.

**Table 3:** Correlations between change scores based on the all GHQ items and the change scores based on the number of items that need to be administered to reach a corresponding level of reliability over 3445 simulated CAT administrations

Theta estimator	Item selection	Prior	Marginal reliability						
			0.96	0.94	0.91	0.88	0.84	0.80	0.75
MLE	UW-FI	–	1.00	1.00	0.99	0.98	0.96	0.93	0.91
MLE	FP-KL	–	1.00	1.00	1.00	0.98	0.96	0.93	0.91
BME	UW-FI	Normal	1.00	1.00	0.99	0.97	0.94	0.91	0.89
BME	UW-FI	Uniform	1.00	1.00	1.00	0.98	0.95	0.92	0.90
BME	FP-KL	Normal	1.00	1.00	0.99	0.97	0.94	0.91	0.89
BME	FP-KL	Uniform	1.00	1.00	1.00	0.98	0.95	0.92	0.90
EAP	UW-FI	Normal	1.00	1.00	0.99	0.97	0.95	0.92	0.90
EAP	UW-FI	Uniform	1.00	0.99	0.98	0.97	0.95	0.93	0.91
EAP	FP-KL	Normal	1.00	1.00	0.99	0.97	0.95	0.92	0.90
EAP	FP-KL	Uniform	0.99	0.99	0.98	0.97	0.95	0.93	0.91

*MLE* maximum likelihood, *BME* Bayesian modal estimation, *EAP* expected A-posteriori estimation, *UW-FI* unweighted Fisher information, *FP-KL* pointwise Kullback–Leibler divergence

For high reliability cutoffs, all items were administered in CAT mode and thus correlations were equal or nearly equal to 1 (i.e. the utility of CAT administration was indeed negligible). As the required reliability got lower this correlation coefficient decreased as the difference between the number of administered items of full-length and CAT modes increased (as well as the utility of CAT). As is clear from Table 3, even for smaller values of reliability correlations were generally high suggesting the close relationship between change scores from full-length and adaptive administration.

## Discussion

Traditionally, applied health and epidemiological survey research has relied on fixed-length questionnaires to measure subjective (mental) health and related constructs. Because most were developed originally as paper forms few researchers experiment with more flexible modes of administration. Fixed-length instruments are popular among researchers because of their familiarity, ease of administration, widespread use and simple scoring (traditionally sum scores). In addition, any comparison of results with studies using the same set of items is straightforward. Thus, there has been little appetite for potentially more optimal administration designs, where technology is needed. Traditional questionnaire surveys are often lengthy in terms of number of items, time consuming to complete, and they may therefore place a considerable burden on patients, some of which might be avoided.

This study provided GHQ-30 calibration (model fit assessment, DIF analysis, and estimation of item parameters) and considerations regarding adaptive administration of GHQ-30 over time in longitudinal studies. The simulation showed that the adaptive administration of the GHQ-30 becomes useful when the required reliability is approximately 0.84 or lower. In that case, a CAT administration would deploy, on average, only half (or less) of the 30 items. Our simulation showed, however, that the utility of CAT depends also on the respondent’s distress level. For individuals with little distress, all, or nearly all items are deployed.

Various *θ* estimators and item selection methods have recently become available in CAT. We selected frequently used options and in terms of efficiency, results suggested similar performance of most of them. However, an informative (standard normal) prior helped to further reduce the number of items, especially for lower reliabilities. Researchers should be cautious when specifying informative priors though, as priors not corresponding with the population distribution may have adverse effects on the number of administrated items [39].

The GHQ-30 was developed as a screening measure to be used by epidemiology, health science and mental health researchers. “Screening” describes two different strategies with different consequences for the usefulness of CAT administrations. In the first strategy, a short test is applied to a large population to identify (groups of) at-risk respondents who might be subject to further (typically longer and/or more expensive) diagnostic tests. For this strategy, screening tests do not necessarily need to be highly precise. Instead they need to be valid, show high correlations with the disorder in question, for example gauged by sensitivity, specificity or predictive values. The reliability of 0.84 mentioned in the previous paragraph can typically be considered as sufficient for such purposes and an adaptive version of GHQ-30 may be an improvement over traditional modes of administration. The second strategy uses the test itself to identify whether an individual respondent may have an unrecognized disorder. For such applications, a highly reliable test is needed to allow for clear decisions about whether an individual is above or below a relevant severity threshold. For this, the confidence interval around the individual severity level or the relevant threshold needs to be small: this decreases the number of cases for which the severity threshold is included in the confidence interval around the individual’s severity level (or the severity level lies within the interval around the threshold, respectively) [3, 38]. A reliability of 0.84 seems rather low for such decisions. These considerations highlight the important role both measurement accuracy as well as validity play in such assessments. Both strategies rest on the assumption that the test is valid in general (appropriate sensitivity, specificity, predictive values).

For both strategies, reliable data on costs associated with the different screening decisions can help to optimise the process. But only the first strategy would allow combining the CAT algorithm with further selection rules, such as choosing the most predictive items, since trading off reliability in favour of validity might be an option, while it would not be for the second strategy.

Our study suggests that the utility of adaptive administration of GHQ-30 items is problematic for the measurement of individual change in longitudinal studies as high reliability is required and all or nearly all items need to be deployed. However, for an assessment of group-level changes in distress, the (random) bias in individual distress change scores cancel out and thus CAT administration may still be a viable option. In addition, the correlations in Table 3 suggest highly similar changes in distress levels (apart from possible linear drifts), captured by either the complete set of GHQ-30 items or the CAT administration, even for low reliability cutoffs (for which considerably fewer items are administered).

An additional potential benefit of CAT administration in longitudinal studies is that respondents measured over time are likely to be exposed to different items (from the same instrument/item pool) at each time they are assessed, whilst keeping the metric of person estimates comparable. This is potentially useful design science, for app-based or web-based data collections [40]. With the recent introduction of mobile devices that increase the frequency of assessment, and perhaps add a new dimension of user-friendliness to questionnaire item delivery, a principled approach to the use of a large item bank could avoid item fatigue or compromise due to over-exposure and thus might help with respondents’ engagement. Obviously, such benefit is suppressed if all or nearly all items are administered. As noted above, CAT algorithms would administer nearly all GHQ-30 items at each occasion to capture individual changes in distress reliably. In summary, CAT administration of GHQ-30 in longitudinal studies which aim to evaluate individual changes would do no harm but may lack utility.

One limitation of this study is that $\theta_{\text{baseline}}$ and $\theta_{\text{followup}}$ estimates based on the complete set of GHQ-30 items are point estimates and thus not true values of *θ*. Therefore $\theta_{\text{change}}$ is not true change and therefore is associated with standard error of measurement. This may limit the size and interpretation of correlations in Table 3. However, the uncertainty accompanied with the point estimates of *θ*_s_ is symmetric and therefore it tends to cancel out in large samples as the one used here.

An additional limitation of our simulation is that we have not considered additional CAT parameters such as item exposure control (meaning whether the researcher wants to restrict or balance any administration profile for the item set or subsets) or the termination criteria (when the CAT stops administering items, e.g. the precision of latent *θ*). In principle, we had no a priori reason with this GHQ item set to control the frequency of any item selection. However, it is worth acknowledging that one concern in CAT is that the standard specifications tend to result in the most informative items being selected too often and the least informative most rarely and therefore item exposure control issues might need to be thought through further in practical applications of adaptive GHQ-30 administrations [4].

As a final limitation, one could argue that the technical resources needed for any CAT application in survey practice might prove to be a barrier to implementation, but while this is certainly a limitation in settings where assessments are not routinely administered on electronic devices, this is not true for surveys. Population surveys usually employ computer-assisted personal interviewing (CAPI) techniques, i.e. electronic devices, to document interviewer—as well as self-rated responses [41]. Their costs were initially discussed controversially [42], but among others the reduced resource use in survey post-processing and the increased quality of the collected data led to today’s wide-spread use of these techniques. In addition, open-source CAT algorithms have become available [10–13]. Their integration into CAPI systems is possible and is still a largely untapped resource [42, 43].

In conclusion, GHQ-30 can be adapted for CAT administration for screening populations. In settings that are usually not interested in individual diagnostic assessments the adaptive presentation can shorten the GHQ-30 considerably and still produce useful estimates of psychological distress for group comparisons. These benefits can be realized in cross-sectional as well as longitudinal surveys. For the assessment of individual changes in distress over time, however, CAT administration may lack utility as nearly all items are administered to reach satisfactory reliability of change scores.

## Appendix: Setup details of CAT simulation

1. *θ* estimators: maximum likelihood estimation (MLE); Bayesian modal estimation (BME); expected a posteriori estimation (EAP).
2. Item selection methods: unweighted Fisher information (UW-FI); pointwise Kullback–Leibler divergence between [*P* ± delta], where *P* is either the current *θ* estimate or a classification bound (FP-KL). For details please see [10].
3. Prior distribution of *θ* (only for BME and EAP): (standard) normal; uniform.
4. Termination criteria (whichever comes first): (a) Precision thresholds (marginal reliability): 0.96; 0.94; 0.91; 0.88; 0.84; 0.80; 0.75 or (b) all items are administered.
5. Initial *θ* starting values: random draws from *U* (−1, 1).
6. Number of items selected for starting portion of CAT: 3.
7. Number of top items from which the function randomly selects next item at initial and middle portion of CAT: 1 (i.e. the most informative item is selected).

## Footnotes

1 For comparison, we also provide model fit for unidimensional model. Baseline: *χ*^2^ = 15133, *df* = 405, *p* < 0.001, CFI = 0.93, TLI = 0.93, RMSEA = 0.10; follow-up: *χ*^2^ = 19806, *df* = 405, *p* < 0.001, CFI = 0.96, TLI = 0.96, RMSEA = 0.12.

2 For the sake of brevity, we only use term reliability in this paper instead of marginal reliability used within CAT context. For details about differences between marginal reliability and traditional, classical test theory view of reliability, please see [36–38].
